# Three-Dimensionally-Printed Polymer and Composite Materials for Dental Applications with Focus on Orthodontics

**DOI:** 10.3390/polym16223151

**Published:** 2024-11-12

**Authors:** Daniela Tichá, Juraj Tomášik, Ľubica Oravcová, Andrej Thurzo

**Affiliations:** Department of Orthodontics, Regenerative and Forensic Dentistry, Faculty of Medicine, Comenius University in Bratislava, 81102 Bratislava, Slovakia; tomasik7@uniba.sk (J.T.); oravcova96@uniba.sk (Ľ.O.)

**Keywords:** 3D printing, crowns, bridges, implants, aligners, removable prostheses, surgical guides

## Abstract

Three-dimensional printing has transformed dentistry by enabling the production of customized dental restorations, aligners, surgical guides, and implants. A variety of polymers and composites are used, each with distinct properties. This review explores materials used in 3D printing for dental applications, focusing on trends identified through a literature search in PubMed, Scopus, and the Web of Science. The most studied areas include 3D-printed crowns, bridges, removable prostheses, surgical guides, and aligners. The development of new materials is still ongoing and also holds great promise in terms of environmentally friendly technologies. Modern manufacturing technologies have a promising future in all areas of dentistry: prosthetics, periodontology, dental and oral surgery, implantology, orthodontics, and regenerative dentistry. However, further studies are needed to safely introduce the latest materials, such as nanodiamond-reinforced PMMA, PLA reinforced with nanohydroxyapatite or magnesium, PLGA composites with tricalcium phosphate and magnesium, and PEEK reinforced with hydroxyapatite or titanium into clinical practice.

## 1. Introduction

### 1.1. Advancements in Additive Manufacturing: Revolutionizing Dentistry and Beyond

**Figure 1:**
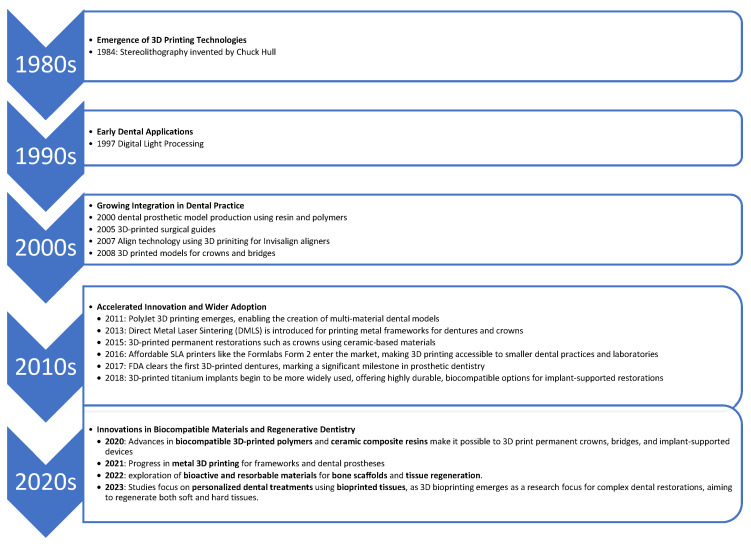
The timeline of 3D printing in dental field [6,7].

Since 1995, 3D printing or additive manufacturing (AM) has been an inextricable part of the mass and individualized production of the most varied construction parts, devices, and even toys (Figure 1). The term stands for the process of subsequent layering of the material used that becomes solid, depending on the technology used. AM introduces novel possibilities for the manufacturing of precise and personalized dental elements, encompassing various forms such as crowns, bridges, implants, aligners, removable prosthetics, surgical guides, occlusal splints, and more [1,2]. There are many advantages of AM compared to traditional production. Additive Manufacturing (AM) is highly cost-efficient. Despite being confined to the printing bed’s total area, AM excels in small-scale production, continually decreasing costs and minimizing material waste and energy consumption. Additionally, AM streamlines inventory management due to its ability to easily recreate and optimize parts [3,4]. Three-dimensional printing is a hot topic within dentistry, just like was shown in the article by Tomášik et al. (2024), which explored the most studied digital technologies within orthodontics, and 3D printing was the second most studied topic in the years 2018–2023 [5]. Ongoing research in 3D printing is crucial as it continually unveils innovative possibilities and enhancements in orthodontics and other fields, leading to more precise and effective healthcare solutions.

### 1.2. Types of 3D-Printed Polymer and Composite Materials

The most common types of materials used in 3D printing include plastics, metals, ceramics, and composites [8]. Most development has been carried out in composite and polymeric materials that are studied in this review. Polymers and composites are both substances consisting of repeated units, but they vary in their composition and structure. Polymers are composed of lengthy chains of repeating molecules known as monomers linked by covalent bonds. These can be either natural, like cellulose and rubber, or synthetic, such as polyethylene and nylon. On the other hand, composites are crafted by combining two or more distinct materials to produce a substance with enhanced characteristics. Typically, one of these materials serves as the matrix and is often a polymer, while the other, known as the reinforcement, possesses different properties. The reinforcement is dispersed throughout the matrix, creating a composite material.

By incorporating metal, ceramic, or nanoparticles into the polymers, composite materials are created. Some examples of 3D-printed composites currently used in the dental sector are as follows:

Metal-reinforced polymers blend the robustness and endurance of metals with the pliability of polymers.

Ceramic-reinforced polymers merge the strength and biocompatibility of ceramics with the flexibility of polymers.

Nanoparticle-reinforced polymers integrate nanoparticles into the polymer matrix to enhance its mechanical properties and biocompatibility. These materials are employed in the fabrication of dental crowns, bridges, and implants.

Some examples of the polymers used for 3D printing in dentistry are Polycaprolactone (PCL), Polymethyl methacrylate (PMMA), Polyether ether ketone (PEEK), Polylactic acid (PLA), polylactic-co-glycolic acid (PLGA), and Ultraviolet (UV) resins [8].

Polymethyl methacrylate (PMMA) stands as the predominant foundational material for dentures, finding its application in the production of temporary crowns, bridges, obturators, retainers, and denture bases. Its notable advantages lie in the straightforward manufacturing process, cost-effectiveness, and stability within the oral environment. To enhance the overall properties of interim prostheses based on PMMA, nanodiamonds in minimal concentrations can be incorporated as reinforcement. The manufacturing of PMMA can be achieved through fused deposition modeling (FDM), and research indicates that stereolithography (SLA) technology is capable of printing PMMA composites with three distinct reinforcements: aluminum nitride, titanium oxide, and barium titanate [8].

Polylactic acid (PLA) is recognized as an eco-friendly material suitable for 3D printing applications [9]. It is compatible with the Fused Deposition Modeling (FDM) technique. The 3D printing process can lead to a reduction in molecular weight and degradation temperature, although it does not significantly alter the semicrystalline structure of the polymer. However, when temperatures exceed 50 °C, PLA can deform, which may limit its applicability in certain 3D printing scenarios. This limitation has prompted the development of hybrid materials that incorporate alternative polymers. For instance, a membrane composed of PLA and reinforced with magnesium demonstrates enhanced charge capacity, corrosion resistance, and cell adhesion, making it effective for guided tissue and bone regeneration (GTR/GBR) applications. PLA is commonly used in the fabrication of orthodontic devices, surgical guides, and temporary crowns and bridges. When PLA scaffolds are combined with nanohydroxyapatite, they can function as effective 3D-printed bio scaffolds aimed at hard tissue regeneration. After being immersed in a simulated body fluid solution for a designated time, PLA scaffolds develop a surface mineral composition similar to hydroxyapatite, composed of calcium and phosphate. The biodegradable characteristics of 3D-printed PLA scaffolds are leveraged to create cell-derived decellularized matrices, thus broadening the use of 3D printing technology in regenerative dental medicine. Overall, PLA is recognized as an optimal material with significant clinical potential [8,10].

Polylactic-co-glycolic acid (PLGA), a copolymer of PLA (polylactic acid) and polyglycolic acid (PGA), is highly regarded as an optimal material due to its ability to biodegrade and its compatibility with living tissues. Created through various methods, PLGA-based composites, when combined with metal reinforcements, offer extensive possibilities for application in dentistry. These composites exhibit versatile characteristics, including promoting cell growth and enhancing antimicrobial properties. The physicochemical attributes of PLGA make it suitable for fabrication using FDM. The ideal parameters for three-dimensional printing vary based on the ratio of PLA and PGA in copolymers, which impacts their molecular weight and end-capping as well as physicochemical properties such as viscosity and heat flow under different temperatures. A higher proportion of PLA in PLGA is recommended for optimal results in 3D printing. PLGA is frequently employed for bone grafts and scaffolds. Three-dimensional PLGA scaffolds have been introduced in the field of tissue engineering. These bioresorbable scaffolds were found to be non-toxic to cells, demonstrated cell proliferation in selected stem cells, and exhibited excellent viscosity. In addressing bone defects, a novel porous scaffold containing PLGA, tricalcium phosphate (TCP), and magnesium powder was created using low-temperature rapid prototyping techniques. It has been confirmed that this newly designed scaffold ensures biosecurity. The in vivo characteristics of 3D-printed PLGA scaffolds have also been evaluated, showing outstanding bone formation and biocompatibility, particularly in the periosteum and in animals with iliac defects. Consequently, biological membranes can be effectively produced using 3D-printed PLGA technology [1,8,11].

Ultraviolet (UV) resins [8,12] consist of a monomer, prepolymer, active diluent, photo initiator, and photosensitizer. When subjected to UV light in the 250–300 nm wavelength range, polymerization occurs rapidly, causing monomers to crosslink and convert from a liquid to a solid three-dimensional structure. The conversion degree is affected by the monomer type, photo initiator, temperature, and light intensity. UV resins are crucial for Digital Light Processing (DLP) and Stereolithographic Printing (SLA) and are used in manufacturing crowns, bridges, surgical guides, dental models, and wax patterns. They offer high curing efficiency, low energy consumption, minimal solvent emissions, and cost-effectiveness. Additionally, the viscosity and curing thickness are influenced by additives like titanium dioxide and tartrazine lake. However, exposure to sunlight can negatively impact the shape and color of SLA-printed products due to the sensitivity of these polymers to UV light [8,12].

Polyether ether ketone (PEEK) is one of the most widely used thermoplastic materials in manufacturing. Recently, there has been a significant increase in the use of fibers to reinforce polymeric materials, aiming to enhance their mechanical properties. As plastic use rises, so does waste production, prompting a trend toward recycling fibers from discarded components for a second life, which can greatly contribute to sustainability efforts. PEEK can also be modified by incorporating hydroxyapatite or titanium to create dental implants. Implants made from PEEK nanocomposites provide notable benefits, including improved bioactivity and mechanical properties [13,14].

### 1.3. Three-Dimensional Printing Technologies in Dentistry

Three primary 3D printing technologies are available, each distinguished by its manufacturing process:

(a) Extrusion-based processes, which utilize solid feedstock materials like filaments or pellets;
(b) Photopolymerization processes involving liquid feedstock materials;
(c) Powder bed fusion processes using powder feedstock materials [15].

**Figure 2:**
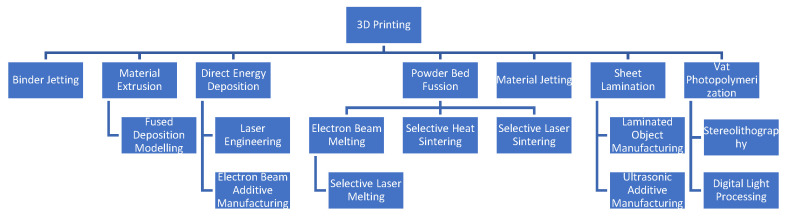
An overview of 3D printing technologies.

The American Section of the International Association for Testing Materials (ASTM) defines technical requirements for various materials and products. It categorizes additive manufacturing (AM) methods into seven types: stereolithography (SLA), material jetting (MJ), material extrusion or fused deposition modeling (FDM), binder jetting, powder bed fusion, sheet lamination, and direct energy deposition (Figure 2) [16,17].

The oldest and most common 3D printing technique in dentistry is VAT photopolymerization (VPP), which forms layered products from a UV-sensitive liquid monomer, which solidifies and polymerizes when exposed to a laser beam. Another widely employed technique is projection digital light processing (DLP). In this method, the device incorporates a microsystem with a rectangular array of mirrors, referred to as a digital micromirror device. When comparing this method with VPP, which features a microsystem with a digital micromirror device. Unlike VPP, DLP allows for faster printing as each layer can be cured simultaneously with a single laser burst, regardless of the number of objects or layer geometry. Both techniques involve three distinct stages: exposure, platform movement, and resin replenishment [11].

Given that dentistry already employs the photopolymerization process in handling composite filling materials, owing to its simplicity and time efficiency, similar benefits extend to 3D-printed models [18]. These advantages include enhanced build resolution, smoother surfaces, robust chemical bonds, and increased mechanical strength [16].

Stereolithography (SLA), the most frequently employed technique in dentistry, involves the polymerization of a liquid resin using an ultraviolet laser [19].

Another method of 3D printing is Material Extrusion (MEX). A definite advantage of this method is its cost-effectiveness and the fact that all materials that can be extruded can be used for production [11]. MEX methods like fused-filament fabrication (FFF) or fused deposition modeling (FDM) are currently not as prominent in the dental industry due to their extended printing durations and limitations in achieving higher resolutions. Among the various technologies considered for the plastics sector, SLA (Stereolithography), DLP (Digital Light Processing), and MJT (Material Jetting) emerge as the most intriguing options, both technically and economically. Additionally, Stratasys’s multi-material 3D printing allows for the simultaneous use of different colors and materials with unique properties in a single build [20].

The aim of this scoping review is to analyze the existing data on 3D-printed polymer and composite materials used in dental applications, examining various features and assessing their clinical acceptance. Secondly, the review aims to map the reasons for the discontinuation of some materials. Additionally, it evaluates safety in the context of recent scientific discoveries regarding the severity of bisphenols.

## 2. Materials and Methods

An online literature search was conducted to identify all research studies relevant to the subject of this paper and published from 2020 to 2023. The search engines PubMed, Scopus, and the Web of Science were used. The database search was carried out on 6 November 2023 using the following search query:

((“3D print”) AND ((“polymer”) OR (“composite”)) AND ((“dental”) AND ((“crown”) OR (“bridge”) OR (“implant”) OR (“splint”) OR (“implant”) OR (“aligner”) OR (“surgical guide”)) AND ((“dentistry”) OR (“implantology”) OR (“orthodontics”) OR (“prosthodontics”) OR (“regenerative dentistry”) OR (“endodontics”) OR (“oral surgery”))

This search query would find articles that discuss the use of 3D-printed materials for manufacturing dental products in all departments of dentistry.

All studies were screened for their title and abstract. Only studies pertaining to the subject matter and only papers written in English were further studied. Based on the title and abstract evaluation, articles, studies, and reviews that were not relevant to the topic of this review were excluded from the search. Similarly, papers written in languages other than English and clinical studies were excluded as well. Papers published before 2020 were excluded in order to yield an as much up-to-date literature search as possible. Only fully published studies relevant to the topic were selected. Searched duplicates throughout various databases were read only once.

## 3. Results

All search conditions were fulfilled in 73 articles. Table 1, Table 2 and Table 3 provide an overview of the most frequently studied materials, 3D printing methods, and dental applications from 2020 to 2023.

As Table 2 shows, the most studied material throughout the searched papers is Polymethyl methacrylate and it was discussed in 15 papers. Table 3 shows that the most frequent 3D printing technology studied in searched papers is stereolithography and it was mentioned in 29 papers. Crowns and bridges are the most frequent dental applications of 3D printing polymer and composite materials throughout the searched papers as it is shown in Table 4.

## 4. Discussion

### 4.1. Contemporary 3D-Printed Polymer and Composite Materials

Contemporary 3D-printed polymers and composites utilized in dentistry are shown in Table 5 as their main characteristics and dental applications.

Resin-based materials can be divided into two main groups:

(a) Acrylic resin-based, which includes Polymethylmethacrylate (PMMA) and Poly ethyl or butyl methacrylate (PEMA)
(b) Composite resin-based, such as urethane Di methacrylate (UDMA) and bisphenol A-glycidyl Di methacrylate (Bis-GMA). Composite resin-based materials are more brittle than acrylic resin-based materials, which might, in turn, cause pulpal irritation from the exothermic reaction produced during the polymerization [50].

There are many materials used for tissue engineering, such as plastics (e.g., PLA, PCL, PETG), ceramics (e.g., hydroxyapatite, zirconia), composites (e.g., graphene, carbon fiber) and biomaterials (e.g., collagen, alginate, chitosan). The advantages of composites are lightweight, high strength, and electrical conductivity. Plastics are flexible and easily customizable, whereas biomaterials are biodegradable and capable of supporting cell growth [32].

Plastic materials can be broadly categorized into two types: thermoplastics and thermosets. In the realm of 3D printing, thermoplastic materials are utilized in two key processes: material extrusion and powder bed fusion. Amorphous thermoplastics, distinguished by their melt properties, are particularly suitable for material extrusion due to their ability to form a highly viscous melt. The nozzle size typically employed for extruding these materials ranges from 0.2 to 0.5 mm. Two prevalent examples of such amorphous thermoplastics used in material extrusion are Polylactide (PLA) and Acrylonitrile butadiene styrene (ABS). Additional instances of amorphous materials applied in material extrusion include Polycarbonate (PC), PC/ABS blend, and Polyetherimide (PEI). In the powder bed fusion process, the most used material is Polyamide 12 (nylon). Other examples of semi-crystalline materials utilized in powder bed fusion include Polypropylene and Polyether ether ketone (PEEK) [3,32].

### 4.2. Material Properties

Basic requirements for 3D-printed restorations can be divided into biological, biomechanical, and aesthetic requirements. They should be biocompatible, non-irritant, have a pleasant odor and taste, and provide a highly polished surface. They must be strong, durable, hard, wear resistant, and able to withstand the functional forces of mastication without fracture or displacement [50].

Dental material is subjected to intraoral thermal changes and occlusal loads that, in the long term, might cause its distortion [21]. Thermosensitive polymers, also known as smart materials, undergo changes in solubility and conformation based on ambient temperature. They find diverse biomedical uses, including tissue engineering, drug delivery, cell culture separation, and nanomedicine, and are compatible with 3D printing. These polymers enable alterations in material properties, such as gelation for topical applications and biodegradable injection scaffolds, stimulated swelling, and hydrogel collapse causing surface property changes. Some of these polymers exhibit thermosensitive behavior within the safe temperature range for the human body. An example is poly-N-isopropylacrylamide (PNI-PAM) and its derivatives. Thermosensitive polymers are divided into two categories: LCST (lower critical solution temperature) and UCTS (upper critical solution temperature). LCST polymers are soluble below their transition temperature but undergo phase separation above it, shifting to a coiled conformation that is energetically favorable. Their solubility relies on hydrogen bonding with water and limited intramolecular interactions. These polymers demonstrate rapid, sharp, and reversible phase transitions in response to temperature changes, influencing the entropy of polymer chain dissolution in aqueous solution. UCTS polymers, on the other hand, undergo phase separation upon cooling, and their transition temperature can be controlled by adjusting copolymer composition, chemical structure, or amino group content. Unfortunately, UCTS polymers do not exhibit biomedical properties as advantageous as those with LCST. Thermosensitive polymers hold immense potential in biomedical applications, offering opportunities for drug delivery tailored to patient circadian rhythms, advancements in gene therapies, and progress in tissue engineering, making them highly promising materials [11].

Oxidative stress is significant in oral inflammatory diseases like periodontitis, linked to genetic polymorphisms in the Glutathione S-transferase (GST) gene family. There was found that periodontitis patients, particularly non-smokers, have altered oxidative stress responses due to GSTM1, GSTT1, and GSTP1 variations. These findings highlight the need for dental materials with enhanced antioxidant properties. Integrating antioxidant-loaded polymers in dental applications, such as implants and scaffolds, could lead to more personalized treatments that address both mechanical needs and the oral biochemical environment [79].

Polymers can be categorized based on their melting temperature and crystalline structure, providing a deeper analysis of the materials used in L-PBF. In terms of melting temperature, polymer powder feedstocks fall into three classifications: high-performance polymers (Tmelting < 260 °C), engineering polymers (Tmelting < 140 °C), and standard polymers (Tmelting < 90 °C). Each sub-class can be further divided based on their molecular structure, either amorphous or semi-crystalline. The categorization according to melting point serves as an approach to group polymers based on their thermal properties. However, a universal processing method cannot be applied to each polymer class due to variations in composition, material properties, and inter-relationships. In this context, the size, shape, and additives of polymer powder significantly impact the density, flowability, spreadability, and, consequently, the processability of the material. The minimum thickness of the powder layer, a crucial parameter in L-PBF, is constrained by the largest particles in the size distribution. Additionally, the width of the powder feedstock size distribution plays a role in influencing the processability of L-PBF [39].

PEEK has an elastic modulus close to that of human bone. This material can be used for manufacturing 3D-printed implants. The most commonly used material for dental implants is titanium. PEEK has similar osteoconductive properties to titanium. Traditional titanium implants allow for a minimal amount of micro-movement, leading to stress concentrations primarily in the apical area of cancellous bone. Nevertheless, even in cases of bone loss, titanium–polyether ether ketone (Ti-PEEK) composite implants demonstrate a broader distribution of stress in the adjacent bone area and prove more effective than conventional titanium implants in minimizing bone resorption (stress shielding). Ti-coated PEEK, showing improved cellular osteogenic differentiation and increased implant osseointegration. PEEK was altered by incorporating hydroxyapatite (HA) for implant applications. Nevertheless, the inclusion of bioactive HA particles in the size range of 2–4 mm can have an adverse impact on the mechanical properties, specifically the tensile strength, of PEEK [14].

Another indication of PEEK in implantology can be temporary dental abutments [14]. Additively manufactured hybrid abutment crowns made from Crowntec (CT) type 3D-printed composite resin and VarseoSmile Crown Plus (VS) 3D-printed hybrid composite resin exhibit lower average gap values compared to subtractively manufactured materials like Brilliant Crios (BC) reinforced composite resin and polymer-infiltrated ceramic network (EN). While differences between materials within each manufacturing method are minimal, the observed variation in the internal fit of hybrid abutment crowns—up to 17.4 µm—may not be clinically significant [61].

The marginal gap of implant-supported 3D-printed definitive composite crowns from SP (Saremco Print Crowntec; Saremco Dental AG, Rebstein, Switzerland) was compared with crowns fabricated by using three different millable materials: reinforced composite BC (Brilliant Crios; Coltene/Whaledent AG, Altstätten, Switzerland), polymer-infiltrated ceramic network VE (Vita Enamic; VITA Zahnfabrik, Bad Säckingen, Germany), and force absorbing hybrid ceramic CS (Cerasmart 270; GC Corporation, Tokyo, Japan). Regardless of cementation, 3D-printed composite resin SP exhibited the lowest marginal gap values [27].

Endosteal implants are prone to stress-shielding and bacterial colonization. Dental restorations should be composed of a material that minimizes stress-shielding and respects mechanical properties, thus minimizing infection risks and expediting patient recovery. Three-dimensional printing parameters suitable for developing dental implants by the FFF technique from PEEK are achieved by the combination of Nozzle Temp: 450 °C, Bedplate Temp: 150 °C, Chamber Temp: 90 °C, Layer thickness: 0.1 mm, and Print Speed: 30 mm/s [40].

PEEK materials used in dental prosthetics are typically divided into three categories: pure PEEK, carbon-fiber-reinforced CF/PEEK, and other PEEK composites. CF/PEEK, formed by incorporating carbon fibers, offers improved mechanical strength and wear resistance. The elastic modulus of CF/PEEK ranges from 3.5 to 58.5 GPa, aligning well with human bone elasticity (13.7 GPa). CF/PEEK is lightweight (density 1.3 g/cm^3^), reducing prosthesis weight for better postoperative stability [14]. PEEK, primarily appearing in jade white and ivory yellow, falls short of meeting aesthetic requirements in dental restorations. To attain a color and translucency resembling natural teeth, resin veneers are frequently applied for surface shading. Clinical acceptability often demands shear bond strength (SBS) values exceeding 10 MPa between PEEK and resin-based composites. Nevertheless, the hydrophobic nature and low surface energy of PEEK pose challenges in establishing a robust and enduring bond [14]. The amorphous polyphenylene sulfone (PPSU) can be a suitable material instead of PEEK [14,73].

### 4.3. Additives of 3D-Printed Composite and Polymer Materials

The technical requirements for various materials and products are established by the American Section of the International Association for Testing Materials (ASTM). This organization classifies additive manufacturing (AM) methods into seven types: stereolithography (SLA), material jetting (MJ), material extrusion or fused deposition modeling (FDM), binder jetting, powder bed fusion, sheet lamination, and direct energy deposition [1,16,17].

Fused Deposition Modeling (FDM) is a significant method of additive processing utilizing thermoplastic polymers to produce components and structures. While various materials can serve as filaments in this technique, PLA holds a prominent position due to its properties and increased environmental awareness in society [1,16].

Laser powder bed fusion is another technique using polymers. Additives change the crystallization kinetics during cooling, induce heterogeneous nucleation, increase laser absorption, and enhance the mechanical properties of polymers. Several materials have been proposed as additives:

- C-based ones (carbon fiber (CF), graphene nanoplatelets (GNP), graphene platelets (GP), carbon nanotube (CNT), multi-wall carbon nanotube MWCNT, and carbon black (CB);
- Oxides (SiO_2_, BaTiO_3_, Al_2_O_3_, K_2_O.TiO_2_, ZrO_2_, TiO_2_, Fe_2_O_3_, Y_2_O_3_, and CaSiO_3_);
- Clays (sulfonated montmorillonite, organo-modified montmorillonite, and hectorite)
- Glass;
- Phosphate (hydroxyapatite and Ca-phosphate);
- Carbonate (CaCO_3_);
- Metals (Al, Ag).

C-based oxides are facilitated to improve the absorbance, mechanical features, and/or electrical conductivity of the built parts. Several types of polymer powder feedstocks are currently available in the commercial market: polyamides (PA12, PA11), polyether ether ketone (PEEK), thermoplastic polyurethane (TPU), polypropylene (PP), and polystyrene (PS). The polymer selection is based on different thermophysical or mechanical properties [39].

The addition of the dye affects the reaction kinetics in acrylic resins. The conversion reaction slows down with the increase in the dye concentration, and conversely, there can be 4,4′-*bis* (N,N-diethylamino) used as an initiator for a much higher conversion rate. Surface-modified sepiolite nanofibers, graphene oxide, and TiO_2_ and SiO_2_ nanoparticles could be used as complementary fillers in epoxy-based resins to enhance the mechanics, particularly sea foam nanofibers, which can increase the tensile strength of resins by 41.4%. Silica (SiO_2_) and zinc oxide (ZnO) increase density and cell proliferation and yield a 2.5-fold higher compressive strength. Epoxy resins filled with aluminum have almost no reduction in mechanical properties and glass transition temperature in humid conditions compared to conventional resins [1].

Incorporating 5% glass fillers and 10–20% zirconia nanoparticles leads to a substantial enhancement in the flexural strength and biocompatibility of the resin material for manufacturing temporary crowns. More precisely, the inclusion of 10%, 20% zirconia, and 5% glass silica by weight significantly amplifies the flexural strength of 3D-printed resins. Biocompatibility assessments indicated cell viability exceeding 80% across all examined groups [44].

PEEK combined with carbon fibers (CFR-PEEK) increased the tensile strength of the composite to 120 MPa and its Young’s modulus to 18 GPa. Coating of PEEK with hydroxyapatite and titanium, the creation of network structures allowing tissue ingrowth, including phosphate–calcium biomaterials, and other modifications are being developed [11]. In nanocomposites, the combination of 0.5% graphene and 3.5% alumina showed an optimal balance, significantly improving wear resistance while maintaining moderate tensile properties [80]. Ceramic biocomposites may contain various reinforcing particles such as TiO_2_, Al_2_O_3_, ZrO_2_, HA, tricalcium phosphate (β-TCP), and calcium phosphate (CaP). A bioactive, thermoplastic, high-performance polymer (Bio-HPP) was obtained by adding 20% of a special ceramic filler to PEEK. Nano-titanium dioxide is a semipermanent antibacterial agent that can generate reactive oxygen species to kill bacteria under ultraviolet light. The highest fracture resistance of the veneers was achieved when a 30% TiO_2_-filled PEEK material was used [14].

The addition of TiO_2_ nanoparticles improved the physical and mechanical properties of 3D-printed denture base material NextDent denture 3D+ light-cured resin (3D systems, Soesterberg, Netherland) in a light pink hue. The best outcomes were associated with adding 0.10 wt%. The addition of 0.50 wt% and more leads to agglomeration of the nanoparticles within the material [63].

Modification of the printable resin by adding zirconia oxide (ZrO_2_) nanoparticles at different concentrations showed superior mechanical properties compared to the unmodified printable resin [12]. The wear resistance of 3D-printed filled resin materials (Pro3dure Medical, Germany, NextDent C&B MFH) was higher than the wear resistance of unfilled resins (Por3dure Medical, Germany and NextDent SG Orange, NextDent, Soesterberg, The Netherlands) [48]. It was found that CROWNTEC (35.530.2 µm) and GC Temp PRINT (16.36.6 µm) had significantly lower wear values compared with Nextdent C&B MFH (86.79.0 µm), though fillers were observed in all materials [23].

The incorporation of calcium silicate into conventional bioceramics like calcium phosphate and calcium sulfate is pursued to enhance the bioactivity, biodegradability, biocompatibility, and drug-loading capacity of these composites, with a specific focus on fostering bone apatite formation and facilitating repair. This approach holds promise as a material for applications in bone regeneration, remodeling, and healing [49].

### 4.4. Dental Applications

There is a wide range of utilizations of 3D-printed dental materials, from prosthodontics, oral and maxillofacial surgery, implantology, and orthodontics to endodontics and periodontology. Besides crowns and bridges, removable prostheses, dental implants, occlusal splints and surgical guides, mouthguards, dental replicas, and periodontal splints were also studied [1,10]. The most used polymers for medical applications are poly-L-lactic acid (PLLA), polyether ether ketone (PEEK), polylactide (PLA), and polymethylmethacrylate (PMMA). Polymer materials and polymer composites meet dental requirements such as specific mechanical and biological properties, corrosion resistance, availability, cost, aesthetics, and relative ease of processing. In addition, the use of polymeric coatings enables increased biocompatibility of bulk materials [11,80].

New temporary resins, partially reinforced with ceramics, have recently entered the market. They can be employed to create inlays, overlays, and single-tooth crowns for both natural teeth and implants. Bonded and tooth-colored restorations provide a time-efficient treatment option for patients. At present, 3D-printed restorations can serve as medium- to long-term provisional solutions. These are particularly indicated for cases involving significant erosions with a loss of vertical dimension, as well as for teeth with an uncertain prognosis or for esthetic rehabilitation [22,74].

PLA is extensively used in medical fields, including regenerative medicine, tissue engineering, implant production, as well as drug delivery. It supports skin and tendon healing and is FDA This material has received approval from the Food and Drug Administration (FDA) for direct contact with biological fluids, making it suitable for medical instruments and equipment manufacturing. Lactide monomers, the building blocks of PLA, exhibit chirality. Through polymerization of L-lactide, D-lactide, D,L-lactide, or mesolactide, one can manipulate the properties of the resulting PLA. To enhance properties like degradation rate, thermal stability, or hydrophilicity, PLA can be blended with other polymers such as polyethylene, polystyrene, polypropylene, or polyethylene glycol. In the case of polylactide, the degradation rate is adjustable, influenced by factors such as polymer composition, molecular weight, crystallinity, pH, additives, production processing, mechanical stress, type of sterilization, and the geometry of the manufactured part approved for direct contact with biological fluids, making it suitable for medical devices. The chirality of lactide monomers allows for the manipulation of PLA properties through polymerization of L-lactide, D-lactide, or D,L-lactide. To improve characteristics such as degradation rate, thermal stability, or hydrophilicity, PLA can be blended with other polymers like polyethylene, polystyrene, polypropylene, or polyethylene glycol. The degradation rate of polylactide can be adjusted based on factors like polymer composition, molecular weight, crystallinity, pH, additives, production methods, mechanical stress, sterilization type, and part geometry [3].

Another indication of 3D printing is the manufacturing of complete removable dental prostheses (CRDPs). A common issue in both traditional and digitally manufactured dentures is the separation of resin teeth from the denture base resin (DBR), representing a type of failure observed in complete or partial dentures [72]. Three-dimensionally-printed removable dental prostheses can require more maintenance visits, adjustment time, and adjustment costs compared to the milled CRDPs [51].

There were artificial teeth for dental prostheses made from four materials based on PMMA. The highest 3D wear was noted in the 3D-printed (PMMA) teeth (PR) teeth (51.05 ± 4.53 mm^3^). This material was compared to three prefabricated commercially available denture teeth: PMMA (Gnathostar, GN), PMMA (SR Orthotyp PE, SR), and Nanohybrid composite (SR Phonares NHC, PH) The limitation of this study is that more precise definition of these materials misses [56]. Polyamide-12 (PA) shows a higher susceptibility than PMMA to discoloration [33].

There was introduced a case of a 16-year-old patient with an impacted permanent tooth. Three-dimensional AM technology allowed the preparation of a new recipient socket with the aid of a surgical replica of the tooth to be transplanted, thus minimizing extraoral time and handling. Med610 material (Stratasys^®^) was used for the 3D printing of the dental replica, and PLA for the printing of the mandibular segment and adjacent teeth [34].

Three-dimensionally-printed models can be also helpful for the diagnostic workup of tumors, surgical planning, and the procedure in oral and orthognathic surgery [35,52]. Also, intraoperative surgical guides and occlusal splints can be truly down-to-earth in this realm [53,68].

Polyether and polyvinyl siloxane (PVS) impressions have been employed to replicate the periodontal ligaments (PDL) with researchers striving to simulate the PDL’s mechanical behavior for reliable laboratory investigations. Replicating the complex structure of the PDL to withstand applied forces poses significant challenges. Establishing standardized PDL thickness with viscoelastic materials complicates the process and demands considerable time and effort. The PDL has an elastic modulus of approximately 850 MPa, which varies with applied loads ranging from 0 to 2.0 N in 0.5 N increments. This stiffness is essential for proper tooth function, as it allows the PDL to absorb and distribute forces during activities like chewing, with different loads affecting its stiffness [77].

### 4.5. Applications in Orthodontics

#### 4.5.1. Three-Dimensionally-Printed Aligners

Invisible aligners are essential in modern orthodontics. In a study from 2022, 3D-printed personalized composite appliances were created using the material Dental LT Clear V2 (Formlabs) for patients with Pierre Robin Sequence through an interdisciplinary approach. The additive manufacturing process was performed with an SLA printer (Form 2, Formlabs, Somerville, MA, USA). Compared to injection-molded and milled acrylates, additive resins often exhibit lower surface hardness, flexural strength, and modulus. Dental LT Clear V2 features an elongation of 12%, flexural strength of 84 MPa at 5% strait, flexural modulus of 2300 MPa, and a Hardness Score of 78 D [65].

**Figure 3:**
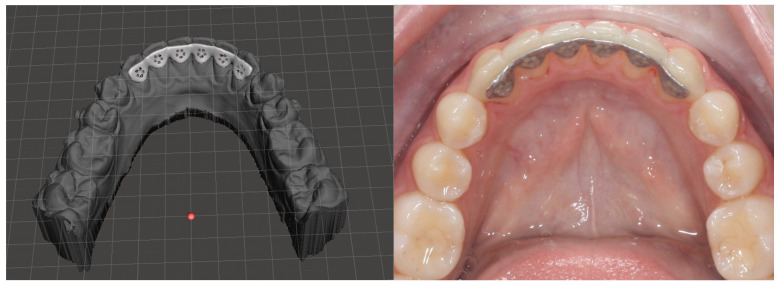
Three-dimensionally-printed fixed retainer from chromium cobalt with modelation before printing.

**Figure 4:**
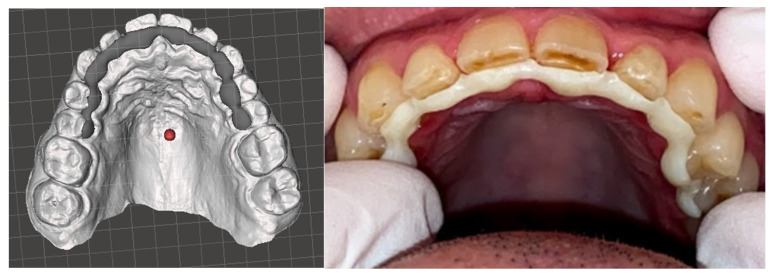
Three-dimensionally-printed fixed retainer from biocompatible resin.

Direct 3D printing allows us to produce clear aligners with precise details and soft edges. These aligners are digitally designed and replicated identically for a complete set of treatment aligners. This method ensures a superior fit, increased effectiveness, and reproducibility. Several clear resins are utilized in 3D printing dental appliances, yet none are specifically designed for 3D-printed clear aligners. In the early part of 2018, EnvisionTEC introduced E-Ortholign, an innovative material tailored for the direct 3D printing of clear aligners. Although categorized as a clear resin, E-Ortholign is positioned as a biocompatible, dimensionally stable, flexible, and robust material primarily intended for the direct 3D printing of the initial aligner, commonly referred to in orthodontics as the “first aligner” or a retainer (Figure 3 and Figure 4). Currently, there is no commercially available and approved photopolymerizable resin specifically formulated for the direct printing of dental clear aligners. However, growing interest in the orthodontic field is evident, particularly in the development of certified biocompatible resins. Despite the absence of established biocompatible resins for 3D direct clear aligners, some experimental trials involving modified resins and post-printing protocols have been conducted on voluntary patients. Nevertheless, clinical outcomes must be validated before scientific publication. Despite advancements and the growing utilization of various 3D printing technologies, there is a scarcity of published research describing the direct 3D printing of orthodontic clear aligners or investigations into appropriate materials for this specific printing process [25]. Direct 3D printing allows us to produce accurate clear aligners that are digitally designed and replicated identically with methods that ensure a superior fit, increased effectiveness, and reproducibility. Several studies have demonstrated the accuracy of digital orthodontic appliances in comparison to their manual counterparts. For example, in a study comparing manual and digital cephalometric workflows, digital methods demonstrated higher accuracy and reproducibility [81]. This correlation supports the argument for digital workflows in orthodontics, where digital methods can substitute traditional manual techniques for improved outcomes.

Materials used in 3D printing for orthodontics encompass a variety of substances, including acrylonitrile-butadiene-styrene plastic, stereolithography materials such as epoxy resins, polylactic acid, polyamide (nylon), glass-filled polyamide, silver, steel, titanium, photopolymers, wax, and polycarbonates. The most common polymers for the manufacture of commercial clear plastic orthodontic aligners are polyester, polyurethane or polypropylene, polycarbonate, ethylene vinyl acetate, and polyvinyl chloride, among others. Within the category of polyesters, both polyethylene terephthalate (PET) and polyethylene terephthalate glycol (PETG)—an amorphous copolymer of PET that does not crystallize—are widely utilized in the manufacturing of clear aligners. This preference is mainly attributed to their exceptional mechanical and optical properties [33].

A novel photocurable resin, TC-85, intended for the direct 3D printing of clear aligners, was compared to the traditional thermoplastic material known as polyethylene terephthalate glycol (PETG). Dynamic mechanical analysis was utilized to assess the mechanical performance of both materials under temperatures of 37 °C and 80 °C. The PETG samples, formed through thermoforming on a standardized model, had an average thickness of 0.41 mm, representing only 54.7% of their original thickness before the thermoforming process (0.75 mm). Conversely, the TC-85 samples produced via 3D printing had an average thickness of 0.56 mm, indicating a 12% increase compared to the intended thickness of 0.5 mm [82].

At the yield point, PETG demonstrated a stress of 44.20 MPa with a strain of 3.92%, while TC-85 exhibited a yield stress of 32.31 MPa with a strain of 4.65% (*p* < 0.01). The elastic moduli of PETG and TC-85 were 1479.54 MPa and 1186.40 MPa, respectively, with PETG showing significantly higher stiffness (*p* < 0.01). Moreover, PETG and TC-85 fractured at approximately 232.93% and 62.55% elongation, respectively [82].

A photopolymer material named Tera Harz TC-85 was designed to address the current limitations of thermoforming aligners made from sheet types. The Tera Harz direct aligner can be directly 3D printed using a 3D printer. It is a biocompatible photopolymer available in two colors, clear and white. TC-85DAC (clear) offers complete transparency, while TC-85DAW (white) provides a combination of durability and aesthetic appeal [33].

There were changes in the mechanical characteristics of custom-made three-dimensional (3D)-printed orthodontic aligners following intraoral aging examined. Sixteen aligners produced with TC-85DAC resin (Graphy, Seoul, Republic of Korea) were utilized, with ten serving as controls (unused) and six retrieved from four patients after one week of use (retrieved group). ATR-FTIR spectroscopy was employed to analyze samples from the control group, while samples from both control and retrieved groups were embedded in resin and subjected to instrumented indentation testing (IIT) to measure force–indentation depth curves. ATR-FTIR analysis confirmed that the aligners were composed of vinyl ester-urethane material. The results from IIT testing revealed no significant differences in mechanical properties between the retrieved and control aligners (*p* > 0.05 for all properties), indicating that the mechanical properties of the 3D-printed aligners remained unaffected after one week of use [83].

#### 4.5.2. Three-Dimensionally-Printed Dental Casts

**Figure 5:**
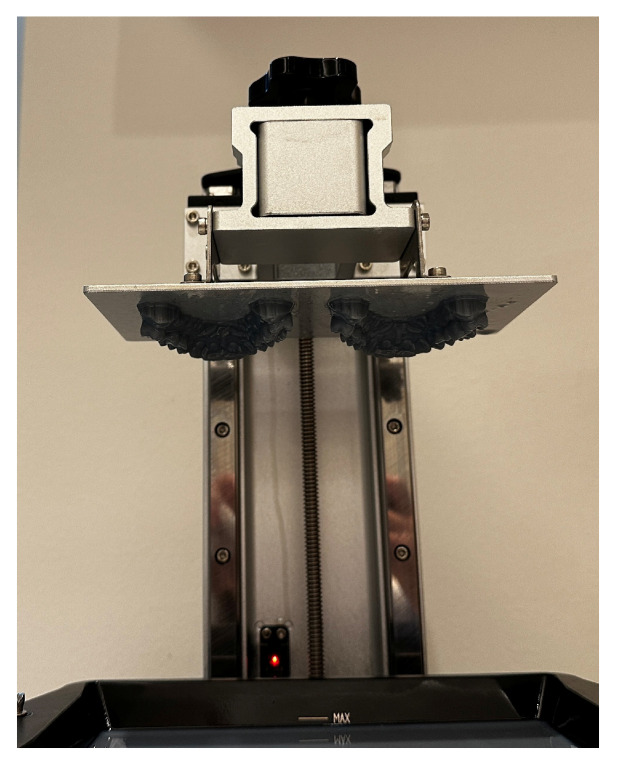
Three-dimensionally-printed dental casts.

Three-dimensional printing replaces the traditional method of casting plasters during the production of dental models. The precision of a 3D-printed dental model significantly influences the outcome of the clear aligner. Utilizing both LCD and SLA technologies can provide a suitable 3D printing approach for creating a dental model (Figure 5) [84].

An instance illustrating the utilization of 3D printing in crafting clear aligners involves the process of 3D printing dental models for molding clear aligners. The incorporation of 3D-printed models represents an initial step in reducing errors and inaccuracies (such as geometric discrepancies) inherent in the traditional method of collecting impressions. Rather than relying on error-prone plaster models that undergo scanning and modeling for creating various alignment stages, opting for digital impression capture and 3D printing proves superior. Thus, the adoption of a clear aligner directly 3D printed for immediate use can eliminate the cumulative errors introduced by analog impressions. Clear aligners can be directly produced in a single processing step through the utilization of one or a combination of 3D printing techniques [25].

There is the study that used the photocurable resin from Shenzhen Anycubic Technology Co., Ltd. (Shenzhen, China) to 3D print dental models from the LCD-SLA 3D printer. The study examined various exposure durations (0.5, 1, 1.5, 8, and 20 s) to create a modeled cube. Consistent parameters such as layer thickness (0.05 mm) were maintained across all cubes in the series. Following printing, the models were rinsed with alcohol. The dimensions of the cubes (L) under different exposure durations were measured using a digital caliper with a precision of 0.01 mm. Cubes with side lengths of 15 and 20 mm were both designed and processed similarly, with an exposure duration set at 8 s to explore the relationship between the actual size of the models and the intended size [84].

Before 3D printing dental models, parameters needed adjustment for utilizing photocurable resin in the 3D printer. A cube with an intended side length of 10 mm failed to form completely when exposed for 0.5 s. Increasing the exposure duration to 8 s enabled complete formation, although the actual side length (L) exceeded the intended length (L0) by approximately 5.5%. After 20 s of exposure, the cube began to take on an irregular shape because the LCD mask could not adequately block light in unwanted areas due to its construction. This led to partial crosslinking of the resin and the formation of thin films that disrupted the intended printing process [84].

The mentioned study also investigated the degree of size variation in models of varying dimensions. They printed cubes with theoretical sizes of 10, 15, or 20 mm using an exposure time of 8 s. Their findings indicated that the absolute difference (L − L0) remained relatively consistent across models of different sizes. However, the relative difference (L − L0)/L0 decreased as the model size increased. This suggests that the effect of size variation is less pronounced when 3D printing larger models. At last, they found out that the exposure time of 1.5 s was most suitable in their system [84].

#### 4.5.3. Three-Dimensionally-Printed Splints

Another possible application of 3D printing in orthodontics is the manufacturing of dental splints. Compared to the analog method, 3D-printed splints for the treatment of temporomandibular disorder (TMD) were very rigid and, therefore, fragile. On top of that the retention was much weaker. On the other hand, the price and working time were lower. The average delivery time for conventional splints is 30 min, while for 3D-printed splints is 15 min [1,2].

NextDent Ortho Rigid print resin was utilized to make occlusal splints on the principle of DLP technology. The printing direction affects the printing time and the amount of material. The least amount of material is needed for the 90° range because it requires fewer supports to hold the splint. The least amount of printing time is required with the 0° printing setting. Although the same STL file was printed, the splint did not fit every time. In most cases, the 0° and 30° orientations were the best [2]. Crowns and bridges printed from commercial printable resin (NextDent C&B Vertex Dental) at 90° orientation using the SLA technique offer higher accuracy (up to 22% error), reproducibility, and efficient material usage. No direct correlation was found between printing layer thickness and elastic modulus or peak stress [64].

### 4.6. Advantages of 3D Printing

From an objective standpoint, the direct 3D printing of aligners presents numerous advantages over the traditional manufacturing process, utilizing the same digital capabilities currently employed in constructing bite splints:

- Digitally designed borders are identically reproduced for all sets of aligners.
- Smooth edges are inherently achieved, eliminating the need for trimming or polishing
- Absence of undercuts due to digital definition
- Enhanced precision in fabrication, eliminating errors introduced during the printing of a 3D molding model and thermoforming stage.
- Improved precision results in better fit and increased effectiveness.
- Customizable intra-aligner thickness, potentially reducing the necessity for attachments that typically compromise the transparency of clear aligners [25].

There are many advantages of 3D printing, as mentioned. Additive Manufacturing (AM) is exceptionally efficient in utilizing resources. Although limited to the total area of the printing bed, AM stands out in small-scale production, consistently reducing costs and reducing both material waste and energy consumption. Furthermore, AM simplifies inventory management by enabling the straightforward recreation and optimization of parts [3,4,71]. The integration of 3D printing technology provides personalized benefits and aids in achieving improved patient outcomes [35]. Three-dimensionally-printed materials showed generally higher wear resistance and smoother surfaces than milled and conventional provisional materials [50].

### 4.7. Limitations of Using 3D-Printed Materials in Dentistry

Nevertheless, there are also some restrictions. Limitations in the realm of 3D printing for dentistry include various considerations. Firstly, devices tailored for dental applications can be relatively expensive, although they typically yield satisfactory final results. Another aspect involves gradations in the Z-direction, where professional-grade printers may exhibit noticeable variations depending on the thickness of individual layers. The impact of layer thickness is significant, with thinner layers resulting in reduced gradation but longer processing times [10]. Three-dimensionally-printed temporary resin dental materials have a lower flexural strength compared with the milled provisional restorations; however, they have higher than conventional provisional restorations [50].

Moreover, there are constraints related to building speed and the size of the building space. The limitations encompass both the maximum achievable build speed and the physical dimensions of the build area. Material properties must match strength, flexibility, and wear resistance. Therefore, it is essential to identify and develop new biocompatible, sterilizable materials that can withstand the harsh environment of the human body. Material restrictions pose challenges, particularly in dental technology, where printers, especially those using photopolymers, limit the range of available resins. This limitation presents a notable disadvantage compared to conventional manufacturing processes [10,32].

Addressing speed issues, emerging technologies such as component detachment methods show promise, but challenges persist, particularly in ensuring the behavior and biocompatibility of the printed devices. Hence, a noteworthy limitation is the scarcity of data on the behavior of 3D-printed devices in the oral cavity. This includes aspects such as plaque formation, elution behavior, and the overall biocompatibility of the printed polymer materials, which currently lack comprehensive information [10].

There are some additives to improve material properties. Still, high loads of additives increased the porosity, which can be linked to unoptimized wetting between additives and polymer matrix. High volume uploading of additives is intended to be improved, further complicating their good dispersion [39].

Accuracy, processing time, and material properties such as ultimate tensile stress, elasticity modulus, yield strength, impact strength, and induced residual stress of printed products are influenced by various factors, such as printer process parameters, material composition, and postprocessing. Build orientation affects material properties, accuracy, and biocompatibility. The final tensile stress and elastic modulus are not significantly affected by the axis and position, but the layout settings have a remarkable effect. Edge-built products show better preference. The compressive strength of the material is better when the printed layer direction is perpendicular to the load direction [1].

The surface properties of 3D-printed objects are significantly altered by the print orientation. However, limitations in cell viability due to the possible leaching of residual uncured photo initiators or the support material were not caused [16]. The problem can be the accuracy of the printing depending on how it is oriented (0, 30, or 90 degrees), the splints can be hard to keep in place, or the printed material is too rigid [2].

An angle of 120° provided greater dimensional accuracy and minimal support surfaces for crowns fabricated by SLA technology to fabricate crowns with various constructive angles. Full-coverage dental restorations fabricated by DLP technology have the highest dimensional accuracy at a tectonic angle of 135°. The other study showed that when the DLP printed three-unit prostheses using two implants in different construction directions with angles of 45° or 60° (corresponding to 135° and 120°), the internal gap was smaller and the fit was higher [1]. Also, the flexural strength increases with a printing orientation of 30° [50]. Two additively manufactured composite resins (Crowntec [CT] and VarseoSmile Crown Plus [VS]) and two subtractive manufactured materials (one reinforced composite resin, Brilliant Crios [BC], and one polymer-infiltrated ceramic network, Vita Enamic [EN]) were used to fabricate standardized screw-retained, implant-supported crowns. The comparison showed that CT and VS had higher volume loss and maximum wear depth than BC, and EN had the highest fracture resistance among tested materials, whereas BC had higher fracture resistance than CT. The differences among tested materials were not significant when the Weibull modulus was considered; however, VE had the highest characteristic strength [75].

Layer thickness also has an impact on material features. There is a rule for SLA: the thicker the layer (the fewer the number of slices), the shorter the construction time; however, the accuracy will be lower. It has been reported that when using SLA technology to print samples with a decrease in layer thickness, the strength of the sample increases. Another study indicated that under the settings of 60 min post-curing time and vertical construction, the optimal layer thickness was 100 μm layer thickness. Considering quality and production time, the most suitable layer thickness for FLM technology is 200 μm [1].

The advancements in 3D printing using plastic waste focus specifically on fused deposition modeling (FDM) and selective laser sintering (SLS) printing methods for carbon fiber composites. The study from 2023 highlight the important role of materials in the 3D printing process, especially regarding the difficulties in producing non-recyclable plastics. Both FDM and SLS 3D printing processes have the potential to reduce plastic waste and create a positive impact on the environment [41]. Theoretically, different 3D printing methods, including fused filament fabrication (FFF), selective laser sintering (SLS) or melting (SLM), stereolithography (SLA), multi-jet photocured polymer process, HP MultiJet Fusion technology, or continuous liquid interface production technology, could be employed for the direct printing of clear aligners. However, considering the unique features of clear aligners and the specific demands for their material characteristics and performance, 3D printing using photopolymerization from clear resin appears to be the most suitable choice [25].

Though 3D printing has the potential to lower the expenses related to manufacturing medical devices, there are still notable costs linked to the technology. These expenses encompass the equipment, materials, time needed for device production, and software costs, which might be challenging for numerous medical facilities. The other challenges of 3D printing are material selection, standardization challenges, accuracy and precision, and regulatory compliance [32].

There were studied three photopolymers proposed for use in PolyJet™ medical 3D printing: Vero Clear RGD810 (VC), Vero PureWhite RGD837 (VPW), and MED610 (M610). These materials were assessed by the MTT assay (3-[4,5-dimethylthiazol-2-yl]-2,5 diphenyl tetrazolium bromide) and Toxodent. No cytotoxicity was proved. This study showed these tests are suitable for evaluating 3D-printed materials for their cytotoxic capacity [3,16,61].

Studies of cytotoxicity and mechanical properties should investigate long-term incubation of materials or testing of modifications or custom-made resins but provide a solid basis for future experiments in these directions [3].

### 4.8. Evolution and Considerations in Dental Materials: From Discontinued Options to Biocompatible Alternatives

Although resin is normally used as a printing material for replicas, some researchers have recently reported the use of titanium or cobalt-chrome alloys to make metallic replicas to prevent the development of deformities during the sterilization process [34].

Over the years, a variety of polymer and composite materials have found application in dentistry, However, certain materials have been discontinued for various reasons, including the following:

- Limited biocompatibility and durability: Some early materials lacked the necessary compatibility with the oral environment, making them vulnerable to adverse reactions in patients. Others exhibited weak mechanical properties, making them prone to wear and fractures.
- Inadequate esthetics: Certain materials, especially early resin-based composites, displayed less-than-optimal esthetic qualities, resulting in noticeable color disparities and poor resistance to staining.
- Difficulties in processing: Some materials posed challenges in terms of processing using conventional dental techniques, leading to inconsistent outcomes and extended chairside time.
- Replacement with superior alternatives: As research advanced and new materials emerged, some older polymers and composites were eventually replaced by more advanced alternatives. These alternatives offered improvements in biocompatibility, durability, esthetics, and ease of processing.

Even the dental use of PEEK is limited at this time, as it requires long-term clinical studies [11]. The other materials that were discontinued in dentistry such as resorcinol-formaldehyde resins (RFRs) and metal-reinforced acrylic resins. RFRs were used for temporary crown and bridge restorations, but they were discontinued due to their irritant features, strong odor, and allergic potential. Metal-reinforced acrylic resins were discontinued due to their poor esthetics, high shrinkage during polymerization, and potential for metal fatigue. They were used for crowns and bridges before. The cessation of these materials underscores the significance of ongoing research and advancement in dentistry. This is crucial for discovering and employing materials that not only yield the best results for patients but also guarantee their safety and effectiveness.

#### Bisphenols

The exploration of dental materials and their compositions is crucial in ensuring the safety and efficacy of medical applications. Among the critical components under scrutiny are bisphenols, a group of chemical compounds widely utilized in various industrial products. Bisphenols are a group of chemical compounds commonly used in industrial materials and products. One of the most well-known bisphenols is Bisphenol A (BPA). These substances are frequently incorporated into polymers and composites utilized in various industries, including medicine, orthodontics, and the dental sector. BPA, despite serving as a key building block for certain plastics and resins, has become a topic of discussion due to its potential negative impact on human health. It is identified as an endocrine disruptor, meaning it can interfere with the hormonal system and pose risks to human health. In practical terms, BPA is often released from polymeric materials, particularly in situations where incomplete polymerization or degradation occurs in the oral environment. This phenomenon has been observed in dental composites and orthodontic materials, raising questions about the safety of using these materials in medical applications. Regulatory measures concerning BPA are gradually tightening. For instance, the European Union banned the production of baby bottles containing BPA in 2011. Stricter limits on BPA usage in the food industry were introduced in 2018. Some countries, such as Denmark, Belgium, and Sweden, have implemented bans on the use of BPA in materials intended for children.

A BPA alternative is Bisphenol S (BPS). That is often used in food and beverage packaging. BPS is also an endocrine disruptor, albeit possessing a lower potency compared to BPA.

Another BPA alternative is Bisphenol F (BPF), which is sometimes used in plastics. Due to a lack of extensive research compared to BPA and BPS, the safety of BPF is not fully understood or established.

The development of new BPA-free materials and efforts to find alternatives to existing polymers with the aim of minimizing exposure to this substance are current trends in research and development. However, it is crucial to note that when replacing these compounds with new ones, thorough safety and biocompatibility assessments should be conducted, especially in the field of medical applications [65].

It is important to note that not all polymer and composite materials used for dental applications contain bisphenols (Table 6). Some materials, such as polycaprolactone (PCL), polylactic acid (PLA), and poly (lactic-co-glycolic acid) (PLGA), are naturally free of bisphenols.

At present, the market for manufacturing 3D-printed teeth is saturated with different varieties, with many introducing new and enhanced features. Nevertheless, there is a clear absence of substantial, evidence-based information regarding the material composition of these products [23]. In the case of 3D-printed resin (NextDent C&B MFH; 3D Systems), interim fixed partial dentures with layer thicknesses of 20 and 50 μm exhibited reduced discrepancies. Consequently, these interim fixed partial dentures might necessitate fewer adjustments during chairside procedures and demonstrate superior marginal adaptation compared to those produced with a 100 μm layer thickness [19].

The biological responses to resinous materials: Keysplint Soft (Keystone Industries GmbH, Stockholzstr. 11, 78,224, Singen, Germany), NextDent Ortho Rigid (3D System. Centurionbaan 190 3769 A V Soesterberg, The Netherlands), Freeprint Splint (Detax GmbH & Co Carl Zeiss str 4, 76,725, Ettlingen, Germany) and the traditional resin Orthocryl (Dentaurum GmbH & Co. Kg, Turnstr 31, 75,228, Ispringen, Germany) were evaluated. Similar biocompatibility, except for Freeprint Splint, which was the most cytotoxic of the four dental resins. The limitation of this study is that there is no information available about the respective compositions of these materials. They were chosen due to their availability and for being some of the most widely used among clinicians [72].

Polylactide possesses impressive capabilities for biosorption, indicating its ability to integrate with host cells and tissues. However, the hydrophobic nature of PLA limits surface interaction with proteins and cells, leading to reduced promotion of cell penetration and the potential for inducing an inflammatory response. Because of these biological and mechanical characteristics, polylactide is not utilized in its pure, unfilled, and unmodified form [11].

### 4.9. Challenges and Opportunities in 3D Printing in Dentistry

#### 4.9.1. Challenges

Material Properties

Challenge: Ensuring the development of biocompatible and mechanically suitable materials for 3D printing in dentistry.

Recommendation: Invest in research for novel materials with improved properties, balancing strength, flexibility, and biocompatibility.

Printing Accuracy

Challenge: Achieving high precision in 3D-printed dental structures, especially in intricate and small-scale applications.

Recommendation: Focus on refining printing technologies, optimizing parameters, and exploring advanced imaging techniques for enhanced accuracy.

Clinical Acceptance

Challenge: Gaining widespread acceptance and regulatory approval for 3D-printed dental solutions in clinical practice.

Recommendation: Collaborate with regulatory bodies, conduct extensive clinical trials, and educate dental professionals on the benefits and safety of 3D-printed dental products. This can be done by modern online e-learning environments [85].

Cost and Accessibility

Challenge: Managing the initial costs associated with implementing 3D printing technology in dental practices.

Recommendation: Investigate cost-effective solutions, promote awareness of long-term benefits, and explore potential partnerships with manufacturers to reduce equipment costs.

#### 4.9.2. Opportunities

Personalized Medicine

Opportunity: Leverage 3D printing to create personalized dental solutions that meet the unique anatomical and functional patient needs.

Recommendation: Prioritize the development of sophisticated software for patient-specific treatment planning. This should include algorithms for customizing implants, prosthetics, and orthodontic devices based on detailed 3D scans and clinical data, enhancing patient outcomes and satisfaction. There should be reinforced biomaterials such as metal-reinforced polymers, ceramic-reinforced polymers, and nanoparticle-reinforced polymers observed for a longer time. Researchers could compare these materials in the same indications, for example, crowns or bridges. These bridges should have the same number of dental pillars and pontics. They should be localized on the same dental segment: lateral or frontal.

Tissue Engineering

Opportunity: Investigate the potential of 3D printing in tissue engineering to fabricate bioactive structures, such as scaffolds for dental implants that promote tissue regeneration.

Recommendation: Establish interdisciplinary collaborations between dental researchers and biomaterials scientists to focus on creating bio-integrated dental implants. Research should target the development of materials that mimic natural tissue properties, thereby improving integration and functionality. There could be some other additional components to current materials.

Digital Dentistry Integration

Opportunity: Seamlessly integrate 3D printing within digital dentistry workflows to optimize treatment planning and execution.

Recommendation: Develop and implement standardized protocols that unify digital impressions, imaging, and 3D printing processes. This integration should include guidelines for data sharing between platforms, ensuring consistency and enhancing efficiency across various dental procedures. There could be some workflow applications for dental offices created. It could be possible to cross the completed steps and see uncompleted ones in this application.

Advanced Imaging Technologies

Opportunity: Employ advanced imaging techniques, such as Cone Beam Computed Tomography (CBCT) and intraoral scanning, to improve the precision of 3D-printed dental products.

Recommendation: Invest in research initiatives that explore the fusion of multiple imaging technologies. Focus on creating comprehensive digital models that accurately represent the patient’s anatomy, facilitating the production of highly precise 3D-printed devices. There should be digital models before and after treatment compared to find the accuracy of the procedure.

#### 4.9.3. Recommendations for Future Research

**Long-Term Biocompatibility Studies**: Conduct a minimum of five-year longitudinal studies on specific 3D-printed materials, such as PLA, PEEK, and nylon, assessing their biocompatibility and mechanical integrity through periodic evaluations of inflammatory responses, material degradation rates, and the incidence of adverse reactions in a diverse patient population.

**Standardization of Protocols**: Create a comprehensive, step-by-step protocol for 3D printing dental materials, including specific recommendations for material types (e.g., type of resin or polymer), printing conditions (e.g., optimal layer thickness of 0.1 mm, nozzle temperature of 220 °C), and post-processing techniques (e.g., UV curing for 30 min at 365 nm) to ensure uniformity in clinical practice.

**Interdisciplinary Collaboration:** Establish formal partnerships with dental schools, material science departments, and engineering faculties to organize annual workshops aimed at addressing technical challenges of 3D printing in orthodontics, such as developing new composite materials that optimize strength and flexibility for brackets and aligners.

**Patient Outcome Studies**: Implement randomized controlled trials that directly compare treatment outcomes of patients using 3D-printed orthodontic appliances versus conventional appliances. Specific metrics to evaluate include treatment duration (e.g., average reduction in treatment time by 25%), patient-reported comfort levels on a scale of 1–10, and effectiveness of tooth movement assessed via digital cephalometric analysis.

**Global Regulatory Frameworks**: Collaborate with the FDA and equivalent bodies in Europe and Asia to draft a set of specific regulatory guidelines for the approval of 3D-printed orthodontic devices, including required testing for mechanical properties (tensile strength, fatigue resistance), and establish a clear pathway for manufacturers to obtain certification for new materials and devices within a 12-month timeframe.

By addressing these challenges and capitalizing on opportunities, the field of 3D printing in dentistry can advance, providing more effective and personalized solutions for dental care.

## 5. Conclusions

In dentistry, 3D printing enables the creation of highly personalized and precise dental prosthetics, crowns, aligners, and implants that perfectly fit the patient’s unique anatomy. This level of customization is particularly critical in dentistry, compared to broader tissue engineering fields where exact anatomical replication may not be as vital. There are many types of 3D-printed polymer and composite materials for dental applications. In the searched articles, crowns and bridges occurred most frequently. In the case of material type, Polymethyl methacrylate (PMMA), Polylactic acid (PA), and Polyether ether ketone (PEEK) were most common. The development of new materials is still ongoing, and the process of enhancing the flexural strength of printed polymers is still in progress. Modern manufacturing technologies show promising future prospects in dentistry based on their remarkable developmental potential.

The materials used in 3D printing for dental applications must meet specific biocompatibility requirements due to constant interaction with oral tissues and fluids. In contrast, other tissue engineering fields may not require the same degree of mechanical resilience and biocompatibility that dental materials demand (e.g., hardness for chewing forces or resistance to wear). While tissue engineering often focuses on regeneration, dentistry applications are more concerned with functional restoration, such as through prosthetics, rather than regenerating entire tissues. However, 3D-printed scaffolds in dentistry can still promote tissue regeneration for bone and tissue healing (e.g., in periodontal therapies).

Three-dimensionally-printed materials replace older methods such as milled and conventional provisional materials. Nonetheless, it is crucial that these materials have the best possible mechanical properties. Material characteristics can be improved by adding other components; however, one needs to carefully consider all aspects of such intentional modifications. Although PEEK’s mechanical strength and wear resistance are increased by adding carbon fibers, they make bonding difficult and lead to frequent failures because of PEEK’s high melting temperature and significant melting viscosity. In the era of modern digital technologies, it appears that much progress in all areas can be achieved using artificial intelligence [86]. Its role is long past providing mere problem analyses and evaluations based on algorithms; it is there to create new ideas and new materials and help find new solutions, such as providing recipes for innovative molecules or setting orthodontic treatment goals, which was not even dreamt of several years ago [5,87,88,89].

## Figures and Tables

**Table 1:** The key differences between polymer and composite dental materials.

Feature	Polymers	Composites
Composition	Single material made up of repeating monomer units	Two or more materials combined to create a material
Structure	Long chains of repeating molecules	Matrix with reinforcement dispersed through
Properties	Varied, depending on the type of polymer	Depending on the properties of the matrix and reinforcement

**Table 2:** Lists the most studied 3D-printed polymer and composite materials as identified by means of the literature search.

Material for 3D Printing	Number of Papers	References
Polymethyl methacrylate	14	[14,17,19,21,22,23,24,25,26,27,28,29,30,31]
Polylactic acid	11	[1,8,11,30,32,33,34,35,36,37,38]
Polyether ether ketone	8	[11,14,30,39,40,41,42,43]
Denture based resin	3	[21,24,44]
Poly (lactic-co-glycolic acid)	2	[8,11]
Polycarbonate	2	[1,38]
Polycaprolactone	2	[8,32]
Carbon fiber reinforced polymer	2	[41,45]
Polyamide	2	[33,37]
Polyamide (nylon)	2	[1,39]
Polyvinyl alcohol	1	[3]
Acrylonitrile butadiene styrene	1	[33]
Acrylic-based resin	1	[37]
Acrylonitrile–butadiene–styrene plastic	1	[33]
Epoxy resins	1	[33]
Polyethylene terephthalate glycol	1	[32]
Thermoplastic polyurethane dental resin	1	[11]
Polylactide-hydroxyapatite nanocomposite	1	[39]
Elastomeric resin	1	[46]
Polymer matrix composite	1	[47]
PCL/PVA composite filaments	1	[48]
PCL/PEG composite filaments	1	[48]
Calcium silicate composite	1	[49]
Calcium sulfate composite	1	[49]
Poly-ε-caprolactone	1	[49]
Nanohybrid composite	1	[23]
Clear resin	1	[25]
Ultraviolet	1	[8]
Metal ionic polymers	1	[15]
Polypropylene	1	[31]
Polyethylene	1	[31]
Undefined	19	[1,4,8,10,13,25,27,42,50,51,52,53,54,55,56,57,58,59,60]

Materials identified in online database search.

**Table 3:** Lists 3D printing technologies studied in searched papers.

3D Printing Technology	Number of Papers	References
Stereolithography	29	[1,4,10,15,19,21,24,25,26,27,30,32,33,36,37,42,46,47,50,54,59,60,61,62,63,64,65,66,67]
Digital Light Printing	20	[1,2,4,10,11,15,19,21,24,27,30,32,37,47,50,54,59,61,68,69]
Fused deposition modelling	18	[1,3,4,15,16,26,30,32,35,36,38,42,43,48,50,59,68,70]
MultiJet fusion technology	11	[4,10,11,13,25,26,27,33,37,54,59]
Selective laser sintering	9	[4,25,30,32,33,37,42,45,50]
VAT polymerization	9	[11,13,17,20,25,33,53,71,72]
Fused filament fabrication	8	[25,33,36,37,40,41,59,73]
PolyJet technique	5	[30,34,50,59,71]
Material extrusion	4	[11,13,28,42]
Powder bed fusion	4	[13,26,39,42]
Binder jetting	4	[4,11,26,32]
Selective laser melting	3	[4,25,33]
Liquid crystal display	2	[59,69]
Electron beam melting	2	[4,32]
Sheet lamination	2	[13,26]
Direct energy deposition	2	[13,26]
Laminated object manufacturing	1	[4]
Inkjet printing	1	[15]
Powder binder printing	1	[33]
Photocured polymer process	1	[33]
Continuous liquid interface production	1	[59]

Three-dimensional printing technologies in searched papers.

**Table 4:** Lists applications of 3D printing of polymer and composite materials in dentistry.

Dental Application of 3D Printing	Number of Papers	References
Crowns and bridges	27	[1,3,8,14,19,21,29,30,31,42,44,45,47,50,54,56,58,60,61,62,64,66,70,71,74,75,76]
Surgical guides	12	[1,10,13,33,35,37,43,52,54,57,66,68]
Removable protheses	11	[1,8,13,24,27,51,54,55,61,66,73]
Aligners	11	[25,28,32,33,36,42,54,55,65,68,69]
3D-printed models	9	[20,25,35,37,42,55,59,68,74]
Dental implants	9	[1,8,11,14,30,32,35,36,42]
Occlusal splints	6	[1,2,52,53,54,55]
Custom trays	4	[1,8,55,76]
Mouthguard	2	[46,48]
Abutments	2	[14,75]
Overlays	1	[22]
Dental replica for autogenous transplantation	1	[34]
Bone tissue engineering	1	[39]
Guided tissue regeneration	1	[11]
Prefabricated teeth	1	[23]
Periodontal ligaments	1	[77]
Undefined	10	[4,15,16,17,41,49,56,63,67,78]

Application of 3D printing in dentistry.

**Table 5:** The main properties and dental applications of 3D-printed materials utilized in contemporary dentistry.

Polymer and Composite Material	Properties	Applications
Polycaprolactone (PCL)	Biocompatible, biodegradable	Temporary crowns and bridges, dental implants, and bone scaffolds
Polymethyl methacrylate (PMMA)	Transparency, strength, durability	Crowns, bridges, dentures, and orthodontic appliances
Polylactic acid (PLA)	Biocompatible, biodegradable	Orthodontic appliances, temporary crowns and bridges, and surgical guides
Poly (lactic-co-glycolic acid) (PLGA)	Biocompatible, biodegradable	Bone grafts and scaffolds
Ultraviolet (UV) resins	High accuracy and precision	Dental models, wax patterns, and surgical guides
Metal-reinforced polymers	Strength and durability of metals with the flexibility of polymers	Crowns, bridges, and implants
Ceramic-reinforced polymers	Strength and biocompatibility of ceramics with flexibility of polymers	Crowns, bridges, and implants
Nanoparticle-reinforced polymers	Improved mechanical properties and biocompatibility	Crowns, bridges, and implants

**Table 6:** Summary of all the polymer and composite materials currently used for dental applications with definition of whether it is polymer or composite material as well as whether it contains any bisphenol.

Material	Classification	Bisphenol Content
Polycaprolactone (PCL)	Polymer	No
Polymethyl methacrylate (PMMA)	Polymer	Yes
Polylactic acid (PLA)	Polymer	Yes
Poly (lactic-co-glycolic acid)	Polymer	Yes
Ultraviolet (UV) resins	Polymer	Yes
Metal-reinforced polymers	Composite	May content bisphenol
Ceramic-reinforced polymers	Composite	May content bisphenol
Nanoparticle-reinforced polymers	Composite	May content bisphenol

## Data Availability

Not applicable.
